# Impact of Extreme Hot Climate on COVID‐19 Outbreak in India

**DOI:** 10.1029/2020GH000305

**Published:** 2020-12-01

**Authors:** Keerthi Sasikumar, Debashis Nath, Reshmita Nath, Wen Chen

**Affiliations:** ^1^ Center for Monsoon System Research, Institute of Atmospheric Physics Chinese Academy of Sciences Beijing China; ^2^ University of Chinese Academy of Sciences Beijing China; ^3^ School of Atmospheric Sciences Sun Yat‐sen University Zhuhai China

**Keywords:** climate change and extremes, COVID‐19, India, population density, temperature and humidity

## Abstract

Coronavirus Disease 2019 (COVID‐19) pandemic poses extreme threat to public health and economy, particularly to the nations with higher population density. The disease first reported in Wuhan, China; later, it spreads elsewhere, and currently, India emerged as COVID‐19 hotspot. In India, we selected 20 densely populated cities having infection counts higher than 500 (by 15 May) as COVID‐19 epicenters. Daily COVID‐19 count has strong covariability with local temperature, which accounts approximately 65–85% of the explained variance; i.e., its spread depends strongly on local temperature rise prior to community transmission phase. The COVID‐19 cases are clustered at temperature and humidity ranging within 27–32°C and 25–45%, respectively. We introduce a combined temperature and humidity profile, which favors rapid COVID‐19 growth at the initial phase. The results are highly significant for predicting future COVID‐19 outbreaks and modeling cities based on environmental conditions. On the other hand, CO_2_ emission is alarmingly high in South Asia (India) and entails high risk of climate change and extreme hot summer. Zoonotic viruses are sensitive to warming induced climate change; COVID‐19 epicenters are collocated on CO_2_ emission hotspots. The COVID‐19 count distribution peaks at 31.0°C, which is 1.0°C higher than current (2020) and historical (1961–1990) mean, value. Approximately, 72% of the COVID‐19 cases are clustered at severe to record‐breaking hot extremes of historical temperature distribution spectrum. Therefore, extreme climate change has important role in the spread of COVID‐19 pandemic. Hence, a strenuous mitigation measure to abate greenhouse gas (GHG) emission is essential to avoid such pandemics in future.

## Key Points

- The survival and growth of SARS‐CoV‐2 in the environment have tighter association with ambient temperature and relative humidity
- Approximately 72% of the COVID‐19 cases in India are clustered in severe record‐breaking hot extreme of historical temperature distribution spectrum
- Extreme climate change has an important role in the spread of COVID‐19 pandemic in India

## 1 Introduction

The world is currently experiencing one of the biggest challenges in the form of global pandemic, i.e., Coronavirus Disease 2019 (COVID‐19), which is caused by the Severe Acute Respiratory Syndrome‐Coronavirus‐2 (SARS‐CoV‐2). The SARS‐CoV‐2 was first reported in Wuhan, Hubei Province, China at the end of 2019 (Huang et al., 2020; She et al., 2020; World Health Organization [WHO] disease outbreak news, 2020; Zhu et al., 2020), then it spreads rapidly to various parts of China and sweeps the entire globe within a span of few months (WHO Situation Reports, 2020). This is now considered as a global crisis leading to an unprecedented situation of lockdown and controls the human mobility. Majority of the affected nations have closed the national borders, shutting down transport system quarantining entire cities and confining the residents at home. Currently, the WHO's risk assessment for COVID‐19 is very high; the total number of confirmed cases exceeds 37 million, and death toll crosses 1 million across the globe (WHO Situation Reports, 2020).

Like other Asian countries, India is at the cusp of deadly transmission of COVID‐19, and therefore, it requires strict lockdown measures to stop any further community transmissions. Despite strict lockdown measures, the current COVID‐19 infection counts shoot up to ~150,000 confirmed cases, with ~86,000 active cases and 4,500 deaths as on 28 May. Maharashtra (Mumbai) alone accounts for 57,000 confirmed cases and 1,900 deaths (https://www.covid19india.org). India is the second largest populated country in the world with an average population density per km^2^ of 464 (https://www.worldometers.info). Respiratory droplets and contact routes are the fastest transmission modes of SARS‐CoV‐2; population density plays a significant role in its transmission (WHO, 2020). Moreover, large scale human migration and transportation can amplify localized outbreaks into widespread epidemics (Colizza et al., 2006). COVID‐19 poses a serious risk to the traveling public because transmission of the emerging pathogens is facilitated by domestic and intercontinental travel (https://www.icao.int/Security/COVID‐19/Pages/default.aspx).

Furthermore, airborne transmission of SARS‐Cov‐2 virus is possible through aerosols with carrier particles size of ≤5 μm diameter (WHO, 2020). Coronaviruses, e.g., SARS‐Cov‐2 virus (100 nm diameters), need watery nuclei to travel through air, or it may bond with carrier particulate matters (PMs) like PM_2.5_ and PM_10_ in atmosphere (Silva et al., 2014; Wong et al., 2010). Pollutants like ozone, SO_2_, NO_2_, and PM have close relationship with infectious disease epidemiology. It is not only the pollution's impact on the immune system; the viruses may interact with the pollutants, allowing them to remain airborne for longer time and helping them make their way to the lungs. The coronavirus may remain infectious for 60 min in air after aerosolization (Pyankov et al., 2018). Based on the current increasing trend in COVID‐19 cases and from the basic understanding of the viral infection spread, there is a strong possibility that SARS‐Cov‐2 may spread through air and thus require adequate control measures for further spreading of the virus (Morawska & Cao, 2020).

In addition, the survival and transmission of Coronaviruses, e.g., SARS‐CoV virus and Middle East Respiratory Syndrome‐Coronavirus (MERS‐CoV‐2012), in the air depend on external factors, e.g., temperature, humidity, and solar intensity (Pyankov et al., 2018; Weber & Stilianakis, 2008). A recent study on COVID‐19 spread in Indonesia showed a significant correlation (*r* = 0.392, *p* < 0.01) between average temperature and COVID‐19 pandemic (Tosepu et al., 2020). In addition, the climate indicators play an essential role in the spread of COVID‐19. A 1° rise in temperature reciprocates ~4.861% increase in the daily confirmed COVID‐19 cases in 122 cities of China (Xie & Zhu, 2020). Evidences from previous studies have indicated the role of meteorological factors in the spread of zoonotic diseases. The MERS‐CoV virus survives at lower temperature (25°C) and high relative humidity (RH) (79%) and remains infectious for 60 min after aerosolization (Pyankov et al., 2018). An increased risk of Hepatitis A was reported around 2 weeks later of extreme hydrological events like extreme daily or weekly rainfall and snow in Spain between 2010 and 2014 (Gullón et al., 2017). Mosquito borne‐zoonotic disease like Dengue showed a delayed effect due to changes in temperature and rainfall, and the relative risks are increasing with temperature and decreasing with extreme rainfall (Chien & Yu, 2014).

Lastly, climate change has significant impact on zoonotic disease epidemiology (Naicker, 2011). Zoonotic infections are transmitted from animal to man (Pappas, 2011), and their interactions are often considered as the potential source of epidemics by generating novel pathogens. Global warming, climate change, and extreme weather events have adverse impact on biodiversity, seasonality, and human incidence of many diseases (Singh & Sachan, 2010). A rise in temperature by 3.3°C is projected by IPCC at the end of the 21st century. It will increase the probability of mosquito borne diseases like the dengue transmission in Dhaka (Banu et al., 2014). A warming at higher latitudes is expected to have implications on Avian influenza virus (AIV) ecology and also on the possibility of virus persistence in the atmosphere (Morin et al., 2018). Increased temperature and precipitation are predicted to have a greatest impact on the transmission of human infectious diseases like tick‐borne diseases, tularaemia, anthrax, and vibriosis in Arctic (Waits et al., 2018). Temperature increases at a rate of 0.2°C/decade and predicted to touch 4°C by the end of 21st century (https://www.ipcc.ch/report/ar4/syr/). In the last decade, the Asian continent has experienced an exceptional number of unprecedented climate extreme events, e.g., severe heat waves in 2015, 2016, 2019, and 2020; the maximum local temperature is rising above 50°C, and the summer of 2013 was the hottest on record in eastern China. This increase in temperature, heat stress, and the frequency and intensity of heat waves may affect animal health by metabolic disruptions, oxidative stress, and immune suppression causing infections and release of the virus (Lacetera, 2019).

In the current manuscript, we analyze the role of population density, temperature, and humidity, underlying the rapid spread and transmission of the SARS‐CoV‐2 virus in India. The extreme climate change has culminated into a tighter association among CO_2_ emission, warming climate, and COVID‐19 pandemic. This research highlights the environmental factors and the conditions which favor rapid spread of SARS‐CoV‐2 at the initial phase (i.e., prior to the community transmission phase) over India. Moreover, this research contributes in the meticulous understanding of anthropogenic climate change impact on the spread of COVID‐19 pandemic in India. We analyze the spatial pattern of COVID‐19‐infected territories and gradual emergence of the epicenters across India. The research results are novel and of utmost importance to understand and contain the spread of SARS‐Cov‐2 virus further. The vivid conjecture retrieved from this research could assist in reassessment of planning of future cities/towns in India, taking into consideration strenuous measures to abate greenhouse gas (GHG) emission and limiting warming further beyond to avoid such pandemics in future.

## 2 Population Density, Ambient Temperature, and RH

**Figure 1:**
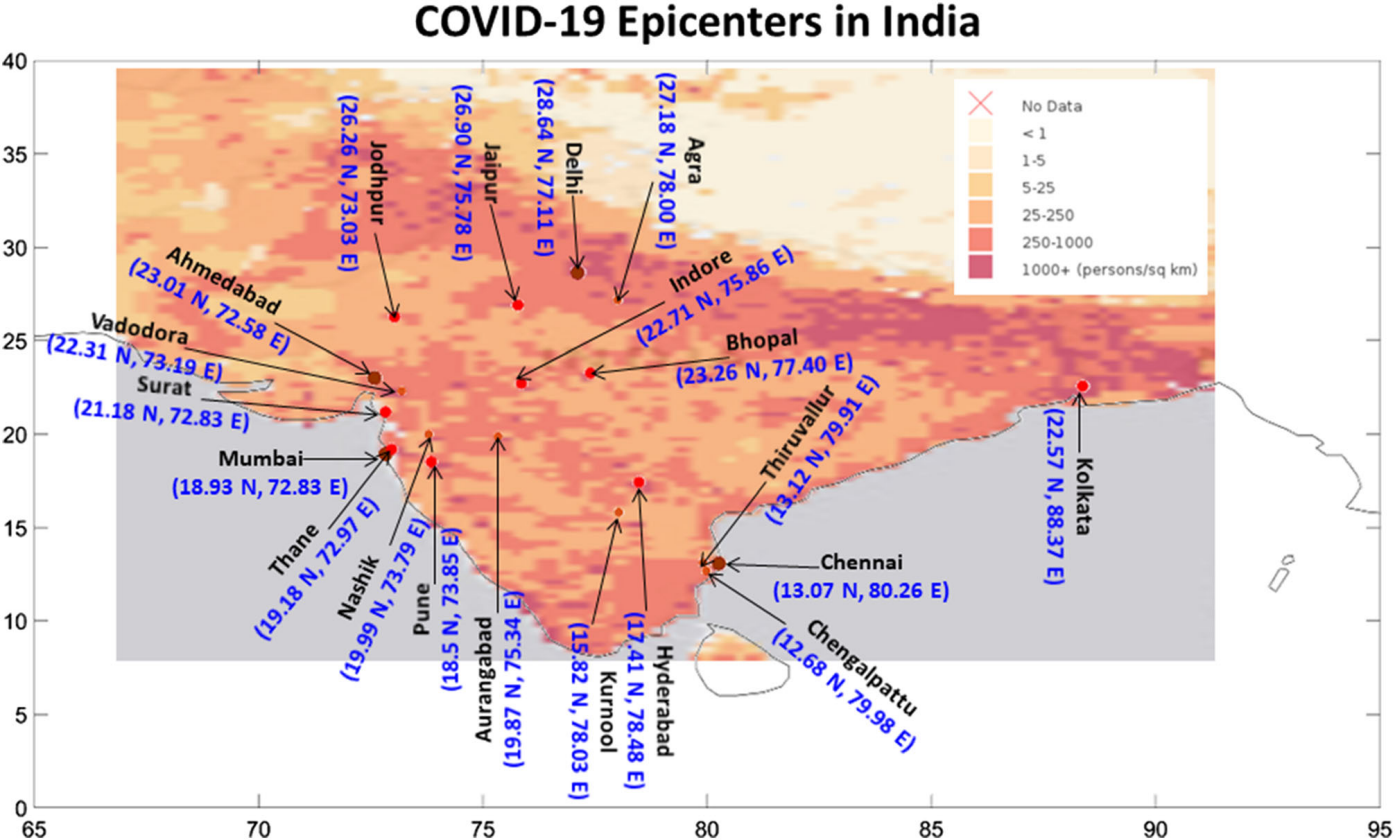
Population density map (color shading) in India and COVID‐19 distribution (dots, updated by 15 May). The epicenters (infection count >500) are marked with dots.

**Figure 2:**
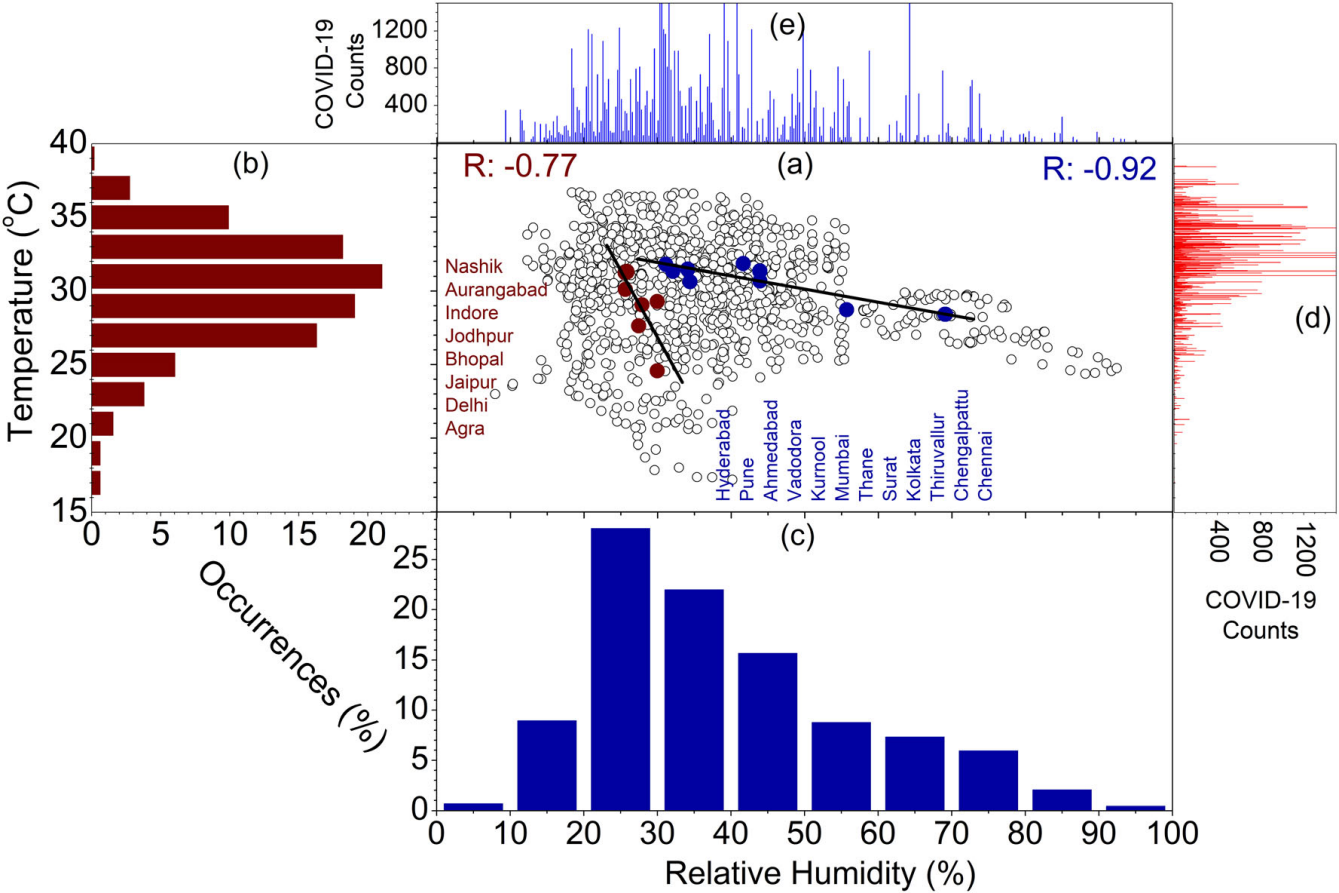
(a) Scatter plot (black dots) of *T* and RH in 20 cities of India. The right panel shows scatter plot (deep red and deep blue dots) of *T*_mean_ and RH_mean_ and linear fits (black line), (b) percentage of occurrences of *T* (2 m) and during the current period (2020, deep red bar) as a function of *T*, (c) percentage of occurrences of RH (1,000 hPa) during the current period (2020, deep blue bar) as a function of RH, (d) total daily COVID‐19 counts as a function of *T* from January to 14 March to 10 May 2020, (e) represents same as (d) but for RH.

Infectious viruses may persist on the contaminated environmental surfaces; however, its duration of persistence and transmission is affected by the climate and weather conditions (Chan et al., 2011). In particular, humidity and temperature are the primary indicators for predicting the influenza epidemics in the temperate and tropical regions (Bedford et al., 2015; Lemaitre et al., 2019; Tamerius et al., 2013). The dried SARS Coronavirus remain viable on smooth surfaces for over 5 days at temperature of 22–25°C and RH of 40–50%, which facilitate its community transmission in the subtropical countries (Chan et al., 2011). In the present study, we sought to investigate whether climate variables like ambient temperature and RH could be the factors in the survival and transmission of COVID‐19 in India.For this study, we select 20 cities of India as epicenters, i.e., the cities with infection counts higher than 500 as on 15 May 2020 (Figure 1, section 5). The cities with highest population density (Figure 1), e.g., Mumbai (28,500 persons/km^2^), Delhi (29,259 persons/km^2^), Kolkata (24,000 persons/km^2^), Chennai (26,903 persons/km^2^), Ahmedabad (12,000 persons/km^2^), Surat (35,000 persons/km^2^), and Hyderabad (18,480 persons/km^2^) (https://censusindia.gov.in), are collocated on the COVID‐19 hotspots in India. Therefore, the higher is the population density, the higher is the proximity between people and the higher is the risk of COVID‐19 infection. In Figure 2a, we plot the scatter diagram (black dots) of *T* (2 m) and RH (1,000 hPa) values over 20 cities and their mean values from NCEP Reanalysis data sets (section 5). During the peak outbreak period (14 March to 10 May) the North Central cities (Nashik, Aurangabad, Indore, Bhopal, Jaipur, Jodhpur, Delhi, and Agra) are clustered within 27–32°C (Figure 2b) and RH values less than 30% (Figure 2c), whereas, in the South and Western cities (Mumbai, Pune, Thane, Ahmedabad, Surat, Vadodara, Hyderabad, Kurnool, Chennai, Thiruvallur, and Chengalpattu), the *T* remains the same but RH is higher than 30%, due to their relative proximity to the coastal regions. The distribution of daily COVID‐19 counts as a function *T* and RH are shown in Figures 2d and 2e, respectively. The majority of the COVID‐19 epicenters are clustered within 27–32°C and 25–45%.

**Figure 3:**
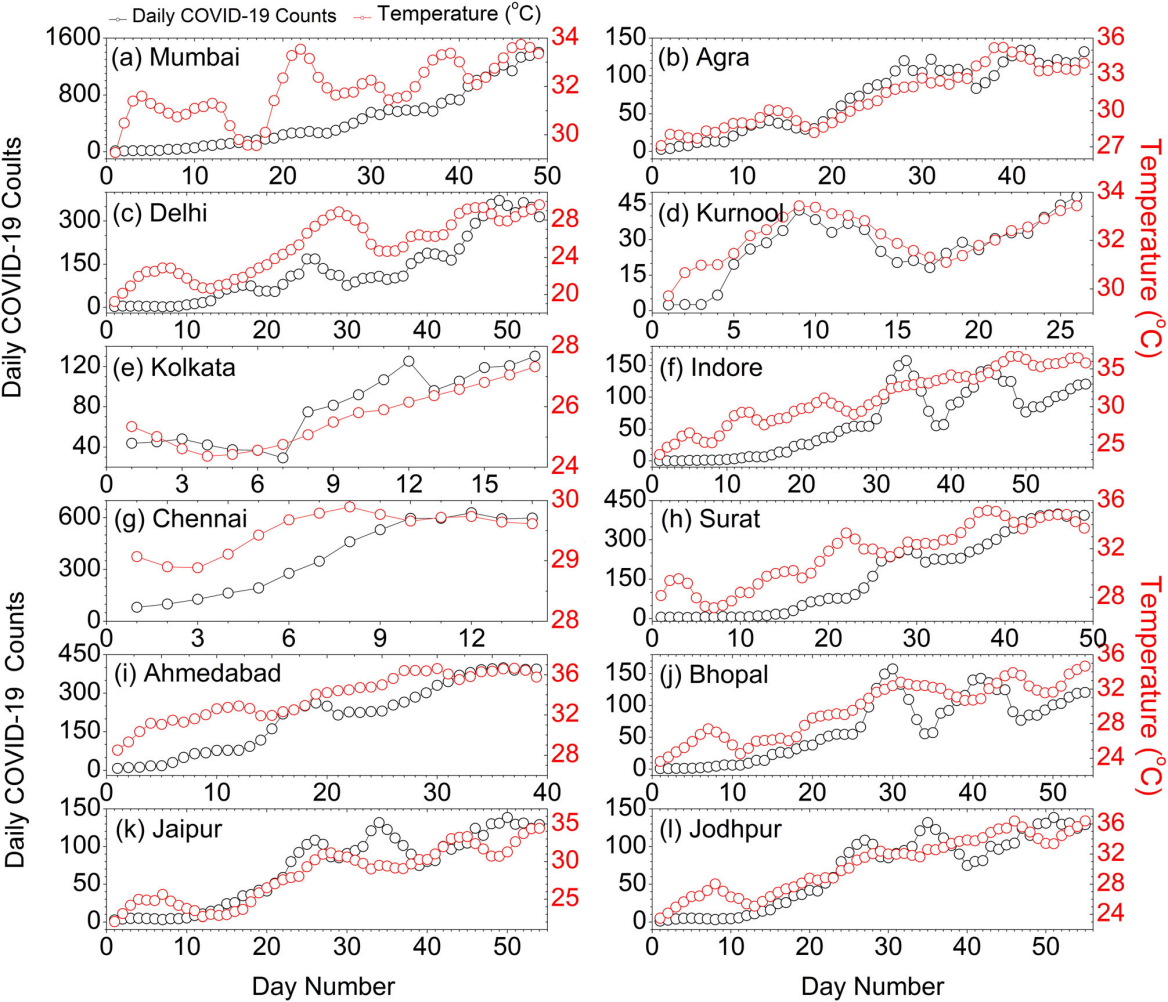
Time series of daily COVID‐19 counts temperature (2 m) over 12 epicenters in India.

**Figure 4:**
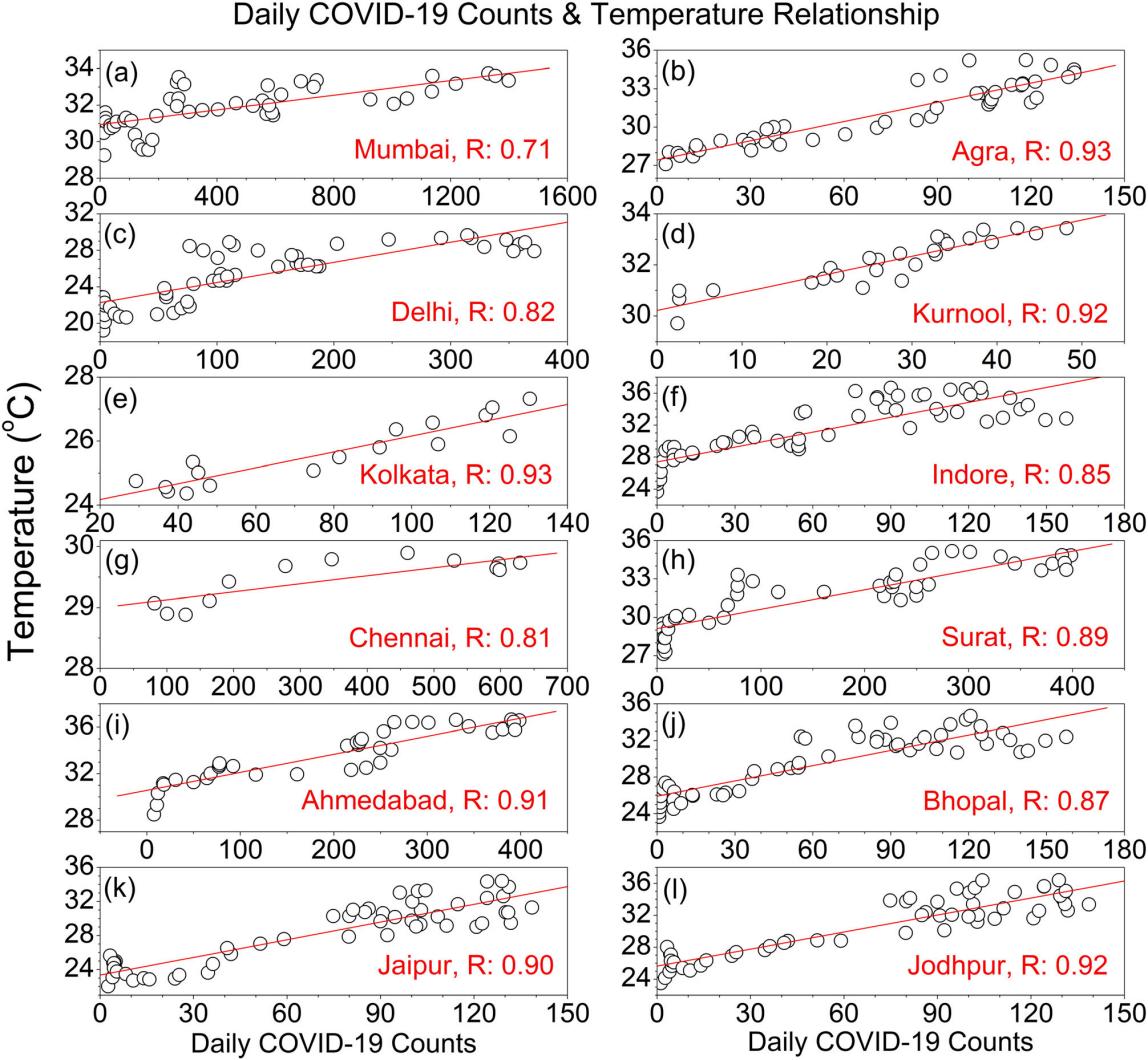
Daily COVID‐19 counts as a function of *T* (2 m) over 12 epicenters in India. The red lines in each subplot indicate the linear fit curve.

In general, survival and transmission of seasonal respiratory viruses have tighter association with ambient temperature. We further analyze the changes in daily COVID‐19 count as a function of *T* for 12 worst affected epicenters, which is shown in Figures 3 and 4. In Figures 3a–3l, we plot the time series of daily COVID‐19 counts and daily *T* (2 m) over the 12 selected cities in India. The daily march of both the variables exhibits high consistency and covariability with *T* (2 m); i.e., daily infection counts are increasing with the rise of ambient temperature. We can observe that at the growing phase of COVID‐19 infection, i.e., prior to the community transmission, the activity of SARS‐CoV‐2 depends strongly on the background meteorological conditions. To further ascertain the linkage between the two factors, we perform the linear correlation analysis between daily COVID‐19 counts and *T* (2 m). The correlation coefficient is higher than 0.9 in Kolkata, Ahmedabad, Jaipur, Agra, Kurnool, and Jodhpur. In Delhi, Chennai, Indore, Surat, and Bhopal, the coefficient ranges between 0.8 and 0.9, whereas it 0.71 in Mumbai (Figures 4a–4l). Therefore, in the growing phase of COVID‐19 outbreak, the temperature covariability accounts for ~85% of the explained variances for first group of cities, 65–80% for the second group of cities, and ~50% for Mumbai. This covariability proves a tighter association of total daily COVID‐19 counts with ambient temperature rise. To obtain a combined profile of temperature and humidity, we employ a best fit linear curve, and the correlation coefficient is −0.77 and −0.92 (Figure 2a) for the both the groups, respectively. This higher correlation coefficient is statistically significant for predicting the COVID‐19 cases and modeling cities or countries based on the environmental conditions. Moreover, based on this knowledge, it is possible to assess the degree of vulnerability of specific cities or countries in different time periods.

It is worthy to mention that apart from high population density and background meteorological conditions, human mobility and hygiene practices are the two critical variables, which have significant impact on the rapid community transmission of SARS‐CoV‐2 (Kraemer et al., 2020). In the current analysis, human mobility has less impact, because during the analysis period, the entire country is in complete lockdown phase, which minimizes the human mobility. However, hygiene practices are not controlled in this analysis, which might have less impact on COVID‐19 transmission in the growing phase (i.e., prior to the community transmission). Higher temperature and population density, though has significant impact on COVID‐19 spread at the initial phase, we cannot rule out the role of other factors like human mobility and hygiene practices.

## 3 CO_2_ Emission, Global Warming, and Climate Change

The Paris Agreement in December 2015 (IPCC, 2015) is committed to combat climate change by holding the global warming below 2°C and to pursue efforts to limit it within 1.5°C. It is widely recognized that limiting the surface air temperature (SAT) rise to 1.5°C, would significantly reduce the risks and impacts of climate change (King et al., 2017). Under the present scenario (business‐as‐usual/RCP8.5), current emissions are tracking slightly above RCP8.5, and significant emission reductions are needed to keep 2°C as a feasible (Perkins, 2006; Rogelj et al., 2016). GHG emissions and global warming have significant impact on the release and transmission of the zoonotic diseases (Ogden & Lindsay, 2016).

**Figure 5:**
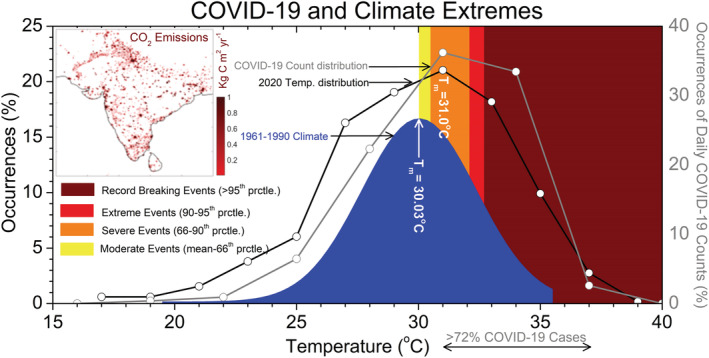
(Left panel) Percentage of occurrences of *T* (2 m) as a function of *T* for historical period (1961–1990, blue shading) and 2020 *T* distribution (black line). (Right panel) Occurrences of daily COVID‐19 count distribution as a function of *T* (gray line). Total emissions (kg C/m^2^/year) from FFDAS V2, average from 1997 and 2015 is shown in top left corner of the plot.

As evident from the Fossil Fuel Data Assimilation System (FFDAS) Version 2 (V2) data, between 1997 and 2015, the global CO_2_ emissions (kg C/m^2^/year) are increasing rapidly (Figure 5); South Asia (India) is accounting much of this rise. This region with higher population density entails high risk of climate change and extreme hot summer and is vulnerable to the infectious diseases (Allen et al., 2017). People residing in densely populated areas possess greater risk of infection and community spread. The higher infection and mortality rates in the densely populated areas will also depend on efficacious healthcare systems. Some demanding parameters such as human mobility and hygiene need to be taken into consideration for COVID‐ 19 spread. The possible connection between population density and COVID‐19 spread is demonstrated in the previous studies, which reported a possible linkage between higher population density and surge in COVID‐19 cases (Bhadra et al., 2020; Coskun et al., 2021). Interestingly, in India, the COVID‐19 epicenters coincide with the emission hotspots and regions with higher population densities, i.e., Mumbai, Delhi, Ahmedabad, Kolkata, Hyderabad, and Chennai.

On one hand, the cities with higher CO_2_ emissions are vulnerable to the COVID‐19 infections, while on the other hand, the survival and transmission of the SARS‐CoV‐2 virus depend strongly on ambient temperature conditions (Figures 3 and 4). Therefore, it is imperative to study the possible connection between emission‐induced climate change and COVID‐19 outbreaks. In Figure 5, we construct the histogram with *T* (2 m) on 20 epicenters in India during the current period (2020) and the historical climatological period (1961–1990). The current period signifies the period from 14 March to 10 May 2020, and the historical climatology signifies the same period mentioned above but for 30 years, i.e., from 1961 to 1990. In addition, we also construct a histogram with COVID‐19 counts over the 20 epicenters (from the date of first reported case to 10 May) as a function of background temperature. We then fit a Gaussian curve on the historical climatological *T* distribution, and the mean value is centered at 30.03°C; i.e., the percentage of occurrences of the distribution is maximum at 30.03°C.

To quantify the shift in mean and the corresponding extremes during the current period with respect to the historical climatological distribution, in Figure 5, we classify the hotter extreme of the historical climatological distribution into moderate hot (mean to 66th percentile), severe hot (66th–90th percentile), extreme hot (90th–95th percentile), and record‐breaking hot (>95th percentile) events; 30.5°C, 32.1°C, and 32.7°C correspond the 66th, 90th, and 95th percentile value of the historical climatological (1961–1990) distribution, respectively. But in the current period, the peak of the distribution shifts toward the hotter extreme by ~1.0°C from its historical mean value and is centered at 31.0°C. The right‐hand side of Figure 5 displays the percentage of occurrences of total COVID‐19 counts over the 20 epicenters as a function of background temperature. Like current period distribution, the Gaussian distribution of the COVID‐19 counts peaks at 31.0°C, i.e., the temperature at which the SARS‐CoV‐2 is more active over the Indian subcontinents. The peak temperature (31.0°C) at which the COVID‐19 counts are maximum lies in the severe extreme of the historical climatological spectrum, and approximately 72% of the COVID‐19 cases are clustered from severe to record‐breaking (66th to >95th percentile) domain of the historical climatological *T* (2 m) spectrum. Therefore, the temperature distribution in the current period is same at its historical climatological value; the percentage of occurrences of COVID‐19 infections should have been significantly reduced. This ~1.0°C rise in temperature over the epicenters can be attributed to the emission induced extreme climate change. The result establishes a relationship between extreme hotter climate and COVID‐19 outbreaks over the epicenters at the initial stage of transmission.

In general, viruses have incredible ability to adapt to the environmental challenges, particularly the temperature changes. Viruses have the capability to block the immune system of the hosts, which is the natural defense system against harmful infections. The increasing CO_2_ emission due to unprecedented fossil fuel burning is the root cause of regional and global warming. A hotter planet may induce excessive heat stress on the zoonotic species, while providing a conducive environment for the viruses to adapt to the newer climate, as well. It could alter the relationship among the infectious agents, host species, and their interactions with the human's immune system. The SARS‐CoV‐2 has the unique capability to adapt with the newer and warmer environment, which is evident from its global transmission patterns (WHO Situation Reports, 2020). Initially, the COVID‐19 infections are restricted to the countries and regions beyond 25°N and temperature ranges between 0°C and 10°C. In the second phase, it spreads to the warmer countries like Sao Paulo in Brazil and Peru in South America and Singapore in the tropical region. In the current phase, India is emerging as the new epicenter for COVID‐19 outbreaks, and the counts are increasing rapidly in the African Continent, as well. Interestingly, India is the closest neighbor to China, Japan, and South Korea, but the count is minimal in the first wave of COVID‐19 infections (January to mid‐March). In the first phase, it is the ambient temperature which possibly inhibits the COVID‐19 to spread in the warmer countries. In the later and current phase, it spreads to the warmer and tropical regions due to higher adaptability of SARS‐CoV‐2 with the ambient temperature, susceptible to higher temperature which is 1.0°C higher than the historical mean climatology. Therefore, COVID‐19 is an obvious outcome and by‐product of extreme climate change induced by greenhouse gas emission.

## 4 Summary and Conclusions

Ongoing COVID‐19 pandemic poses serious threat to the public health worldwide, due to faster transmission and contagious nature of SARS‐CoV‐2. India, being the current epicenters, we have selected 20 worst hit cities having counts higher than 500 (as on 15 May). Regions of higher population density are vulnerable to the COVID‐19 infections worldwide. The survival and growth of SARS‐CoV‐2 in the environment have tighter association with ambient temperature and RH. In India, the SARS‐CoV‐2 is most active at temperature ranges between 27°C and 32°C and humidity between 25% and 45%. Moreover, the daily COVID‐19 counts have higher covariability (~65–85%) with local temperature; i.e., spread and growth of SARS‐CoV‐2 depend on local temperature rise. The combined temperature and humidity spectrum is highly significant for predicting the COVID‐19 cases and modeling cities or countries based on the environmental conditions by assessing the degree of vulnerability at the initial phase (i.e., prior to the community transmission phase). It is known that the zoonotic viruses and its spread depend strongly on background meteorological conditions. Warming‐induced climate change has significant impact on zoonotic disease epidemiology and its transmission from animal to man. Global warming, climate change, and extreme weather events have adverse impact on biodiversity, seasonality, and human incidence of many diseases. A projected rise in surface temperature by 3.3°C in IPCC models at the end of 21st century will increase mosquito activity and a substantial increase in dengue and AIV activity. In the current analysis, we construct the histogram with *T* (2 m) on 20 epicenters in India during the current period (2020) and the historical climatological period (1961–1990). Moreover, the distribution of the COVID‐19 counts over the 20 epicenters peaks at 31.0°C, which is ~1.0°C higher from its historical mean value. In the climatological *T* distribution spectrum, this peak temperature (i.e., 31.0°C) belongs to the severe extreme, and 72% of the COVID‐19 cases are clustered between severe and record‐breaking extreme range.

From this analysis, we establish the fact that SARS‐CoV‐2 is highly sensitive to the regional warming‐ and emission‐induced climate change, which is evident from the fact that epicenters are collocated on CO_2_ emission hotspots. Therefore, the higher the emission, the higher is the warming and the higher is the probability of COVID‐19 transmission at its initial phase. Under current scenario, the health risk is serious for the global community, which is an obvious outcome of anthropogenic climate change due to greenhouse gas emission. Therefore, extreme climate change has an important role in the spread of COVID‐19 pandemic, and thus, strenuous mitigation measure to abate GHG emission and limiting warming further beyond is an urgent need to avoid such pandemics in future.

## 5 Data and Methods

For our study, we select the COVID‐19 pandemic epicenters or cities in India exceeding the infection counts of 500 by 15 May 2020. The 20 have satisfied the above criteria that are shown in Figure 1. It includes Mumbai, Thane, Pune, Nashik, Aurangabad, Ahmedabad, Surat, Vadodara, Delhi, Agra, Jaipur, Jodhpur, Indore, Bhopal, Hyderabad, Kurnool, Chennai, Chengalpattu, Thiruvallur, and Kolkata. The daily and cumulative COVID‐19 counts data available online (https://covid19.who.int/ and https://coronavirus.jhu.edu/).

For daily air temperature (*T*) at 2 m and RH (1,000 hPa) data, we used the NCEP Reanalysis product (https://psl.noaa.gov/data/timeseries/daily/) over the selected locations, spanning from 14 March to 10 May 2020 (peak date for worldwide COVID‐19 counts). For our analysis, we have chosen the date when first confirm case was reported in the individual cities. Two‐meter temperature (2 m) is temperature at the height of 2 m above Earth's surface. RH at 1,000 hPa is the percentage of the maximum amount of water vapor that the atmosphere can hold at a given temperature (saturation). For daily climatology, we used the 30 years of NCEP data from 1961 to 1990. We applied a 5‐day smoothing on each variable to obtain the background environmental condition, considering the COVID‐19 incubation period of ~5 days (Guan et al., 2020).

The population density data in Figure 1 can be downloaded from the NASA Socioeconomic Data and Applications Center (SEDAC) (http://sedac.ciesin.columbia.edu/data/collection/groads/maps/gallery/search). FFDAS version 2.0 is a data product that estimates CO_2_ emissions from the combustion of fossil fuel for the years 1997–2015 on a global, 0.1° × 0.1°, hourly grid available from the Purdue University. It estimates fossil fuel CO_2_ emissions by constraining elements at the grid cell level with national statistics on fossil fuel CO_2_ emissions, remotely sensed nighttime lights, population data, and information on the world's power plants.

Statistically, we classify hot extremes in the historical *T* (2 m) distribution curve using the percentile method. Record‐breaking hot ≳ 95th percentile, extreme hot = 90th–95th percentile, severe hot = 66th–90th percentile, moderate hot = mean to 66th percentile.

## Conflict of Interest

The authors declare no conflicts of interest relevant to this study.

## Data Availability

NCEP Reanalysis data are available online (https://psl.noaa.gov/data/timeseries/daily/). The map of Global Population Density (2020) and Global annual PM2.5 Grids from MODIS, MISR, and SeaWiFS Aerosol Optical Depth (AOD) were downloaded from the NASA Socioeconomic Data and Applications Center (SEDAC) (http://sedac.ciesin.columbia.edu/data/collection/groads/maps/gallery/search), accessed on 1 May 2020.
